# Results of Pasteur’s Inoculation

**Published:** 1886-03

**Authors:** 


### RESULTS OF THE CONTINUATION OF PASTEUR'S INOCULATION FOR RABIES.

The Paris correspondent of the *Lancet* writes that the young shepherd (Jupille) referred to as being still under observation, and upon whom M. Pasteur has since ceased his inoculations, has returned to his native village. His room is now occupied by a lad of fourteen, who is said to have been bitten by a mad dog at Bordeaux, and two other children, also bitten by a mad dog, have been forwarded to Paris by the mayor of Roubais, to be subjected to M. Pasteur's treatment for hydrophobia. The persevering and zealous experimenter will soon have his hands full, and the confidence in his remedy is such that people come from great distances to consult him, without even taking the trouble to find out whether the dogs that bit them were really rabid or not ; so that M. Pasteur will, under the circumstances, have some difficulty in selecting his cases. Owing to the favorable results obtained by M. Pasteur, it is proposed to organize a special staff in different localities for the carrying out of his method, and that all medical men should be instructed as to the manner of its employment. The only question seems to be satisfaction about the persons having been affected by rabies at all. It is not every dog that bites a person that has rabies, nor every dog that froths at the mouth and acts strangely that has anything like rabies; but it is such a terrible thing to be bitten by a dog that has it, that every one bitten, doctors for that terrible disease. It will take a good many well-proved cases to prove Pasteur's remedy satisfactory to the medical profession or the world who watch and think on the subject.

Considerable excitement has been raised in Arlington, N. J., by the report of a case of hydrophobia in that place—a curious case apparently, and one which we should hope might be more effectual, if possible, than his. The case was in the Catholic Protectory at that place. William Klee, a ten-year-old German lad, was sent to the Protectory some months ago from Paterson as an incorrigible truant. He was allowed to visit his mother during the holidays, and was bitten in the hand on January 2, by a mongrel dog. On January 17 the boy went back to the Protectory in Arlington and on Friday night, twenty-one days after being bitten, he was seized with convulsions in which he manifested every symptom of hydrophia. He tried to bite himself and his attendants, and had to be held down in bed. In the intervals between the spasms he complained of great pain in the head and back and an almost unbearable choking sensation in the throat. The convulsions continued all day Saturday.

On Sunday one of the Sisters in charge suspected that the boy was suffering from hydrophobia, as he was snarling and barking like a dog. On Friday night Father Curran, the superintendent, who is noted for his skill in medicine, had given Klee a dose of bromide of potassa, and on Saturday the Sister had given him a big dose of castor oil. On Sunday Father Curran decided on giving the patient a severe sweating. Dr. Exton, the house physician, called at two o'clock in the afternoon. Klee's pulse was very irregular, and the pupils of his eyes were dilated. At half-past two P. M. he was placed in a vapor bath, where he was kept for half an hour. The temperature was 212 degrees, and the boy cried with pain caused by the extreme heat.

At the end of the half hour he was removed from the bath, wrapped in dry blankets and put to bed. He continued to sweat for four hours so profusely that the perspiration discolored the blankets. There has been no return of the attack since.

---

**The** President of the Los Angeles Medical Society has appointed Dr. Elizabeth A. Follansbee Chairman of the Board of Censors for 1886, for that county.

